# Pulmonary Epithelial-Myoepithelial Carcinoma

**DOI:** 10.1155/2022/4559550

**Published:** 2022-10-11

**Authors:** Lingru Chen, Ying Fan, Hongyang Lu

**Affiliations:** ^1^Zhejiang Key Laboratory of Diagnosis & Treatment Technology on Thoracic Oncology (Lung and Esophagus), Cancer Hospital of the University of Chinese Academy of Sciences (Zhejiang Cancer Hospital), Hangzhou 310022, China; ^2^Department of Thoracic Medical Oncology, Cancer Hospital of the University of Chinese Academy of Sciences (Zhejiang Cancer Hospital), Hangzhou 310022, China; ^3^Institute of Basic Medicine and Cancer (IBMC), Chinese Academy of Sciences, Hangzhou 310022, China; ^4^Wenzhou Medical University, Wenzhou 325035, China

## Abstract

Pulmonary epithelial-myoepithelial carcinoma (P-EMC) is an exceptionally rare subtype of salivary gland lung tumor originating from tracheobronchial glands. P-EMC is a biphasic tumor consisting of an inner layer of epithelial cells and an outer layer of spindle-shaped, clear-cell-like myoepithelial cells. Bronchial obstruction symptom is the main clinical characteristic for P-EMC. Because its clinical and imaging characteristics are highly similar to other types of non-small-cell lung cancer (NSCLC), it is easy to cause missed diagnosis and misdiagnosis. The diagnosis is mainly based on the pathology and immunohistochemistry with an inner layer of epithelial cells immunoreactive for cytokeratin and an outside layer of myoepithelial cells immunoreactive for S100 protein (S-100) and smooth muscle actin (SMA). Therefore, positive for cytokeratin, S-100 and SMA can assist in the diagnosis. Although in general, P-EMC is a low-grade malignant neoplasm, it may occasionally recur and metastasize. The optimal method for P-EMC treatment has not been established, and surgical resection is still the main clinical method. Radiotherapy and chemotherapy have been shown not sensitive for P-EMC treatment, whereas targeted therapy and immunotherapy have not evaluated in clinical practice. This review focuses on the pathological characteristics, molecular characteristics, diagnosis, treatment, and prognosis of P-EMC.

## 1. Introduction

Pulmonary epithelial-myoepithelial carcinoma (P-EMC) is an exceptionally rare salivary gland-type tumor (SGTT), which accounts for 0.1-1% of all primary lung carcinomas [1, 2]. Among the SGTTs, the most common histological types are adenoid cystic carcinoma (ACC) and mucoepidermoid carcinoma (MEC), while mixed tumor (ACC and MEC) and EMC are less common [3]. In 1972, EMC was first described by Donath et al. [4], and this carcinoma was subsequently defined by the WHO as “a malignant tumor composed of variable proportions of two-cell types, which typically form duct-like structures.” The duct-like structure is characterized by a biphasic pattern, with an inner layer of epithelial cells immunoreactive for cytokeratin and an outside layer of myoepithelial cells immunoreactive for S100 protein (S-100) and smooth muscle actin (SMA) [5]. P-EMC originates from the submucosal tracheobronchial glands, which are the equivalent of the minor salivary glands; but EMC is rarely found in the respiratory tract [6]. In general, P-EMC lacks typical epidemiological and clinical characteristics. Therefore, in view of the biphasic structure, the diagnosis of P-EMC is usually based on microscopic morphologic examination of biopsy specimen in combination with immunohistochemistry (IHC) examination. Although it is generally considered as a low-grade malignant neoplasm, occasional recurrence and metastasis have been reported [7]. However, the current reported cases of P-EMC are rare, and little is known about the molecular characteristics of this malignancy. The treatment of P-EMC is mainly based on surgical resection, while the effect of radiotherapy and chemotherapy has not been determined. In addition, the prognosis is also not well-established for P-EMC. Here, we review the epidemiological features, clinical characteristics, pathology, immunohistochemistry, diagnosis, treatment, and prognosis of P-EMC.

## 2. Epidemiological Characteristics and Clinical Characteristics

Because of its rarity, the epidemiological features of P-EMC have not been well defined. EMC accounts for less than 1% of all salivary gland tumors [8]. A large proportion of the patients are middle-aged people, but the youngest case reported is 7 years old [9]. From the current literature, female patients account for the majority of P-EMC cases. So far, in the reviewed cases, only ~50% of the patients are smokers, so it seems unlikely that smoking is the cause of this disease [7]. Epidemiological studies of more cases are required to determine whether smoking is a risk factor for P-EMC.

In general, P-EMC arises from submucosal tracheobronchial glands [6]. The existence of tumors in the peripheral lung tissue suggests that P-EMC might originate from primitive cells [10]. In the cases of bronchial obstruction, the patients showed varied symptoms of airway obstruction including productive cough, chest pain, shortness of breath, repeated bouts of bronchopneumonia, recurrent chest infection, blood-tinged sputum, hemoptysis, and other symptoms such as fever, weight loss, and enlargement of lymph nodes. The tumor is mainly located in the central airway within the segmental bronchi of the most patients with these symptoms. Some patients were asymptomatic at first, but they would also have bronchial obstructions in the end due to obstruction of the subsegmental bronchus [11]. However, some patients showed no symptoms in daily life, but were discovered accidentally during physical examinations. P-EMC is a typically indolent cancer, but it is potentially malignant, and metastasis may occur [5]. The reported metastasis sites include hilar and subcarinal lymph node, skull, bone of left lower limb, bilateral pulmonary, and chest wall [12–16].

In Table 1, we summarize the epidemiological and clinical characteristics of P-EMC based on published studies. These cases of P-EMC include 40 females and 25 males with an average age of 56 years, among which only 14 patients had a history of smoking [5, 6, 7, 9, 11–42]. Therefore, P-EMC mainly occurs in middle-aged and elderly female patients, and it is not clear how smoking is related to the incidence of P-EMC. The main location of P-EMC is endobronchial mass and the main clinical feature is bronchial obstruction [5, 6, 7, 9, 11–42]. The clinical characteristics may be closely related to the location of the tumors.

### 2.1. Imaging Examination

Imaging examinations are an important means for discovering P-EMC, including X-ray and chest computed tomography (CT). According to the literature, the X-ray chest film of the tumors shows no characteristic signs. Because P-EMC predominantly appears as endobronchial mass, CT is the most common and basic imaging examination method for P-EMC. As shown in Table 1, 53 cases were presented as endobronchial masses distributed in the central airway within segmental bronchi, 11 cases were located in pulmonary parenchyma, and 6 cases were presented as intraparenchymatous mass without obvious relation to bronchus [5, 6, 7, 9, 11–42].

Some studies have reported active fluorodeoxyglucose (FDG) uptake in P-EMC [16, 29]. However, in a recent study, Nakashima et al. [11] found that, in most cases, abnormal FDG uptake was not detected by FDG-positron emission tomography/computed tomography (FDG-PET/CT) scans.

There are different growth patterns and imaging manifestations in P-EMC. In general, most of the reviewed cases presented an image of a well-circumscribed and polypoid solid mass without hilar or mediastinal lymph nodes enlargement. In some cases, irregularly-shaped tumors on CT scans have been reported, such as a cavitary lesion and an abnormal soft tissue mass with slightly lobular borders [33, 36]. So far, there were no typical and characteristic radiographic features were found in P-EMC.

In summary, P-EMC mostly occurs in endobronchial masses, the most common method of imaging examinations is CT. A well-circumscribed and polypoid solid mass without hilar or mediastinal lymph nodes enlargement is the most common imaging sign. It is worth noting that the imaging features of tumor distribution and morphology are uncertain because of the limited cases of P-EMC. At the same time, bronchoscopy also provides a means for a definitive diagnosis.

## 3. Pathology and IHC Examinations

Grossly, the P-EMC tumor have varied sizes and are usually isolated with a well-defined, encapsulated polypoid mass. However, rare exceptions such as papillary and cauliflower-like neoplasms were also reported [37, 42]. P-EMCs generally appear as solid mass with white, white-greyish, light-yellow, or red color. Necrosis and marked mitosis are rarely seen for P-EMC [36]. Microscopically, there is a biphasic structure for P-EMC in histology. There are three distinct subtypes of the tumor: the first has a dual ductal component, which is a feature of salivary gland tumor; the second is present with a predominant solid component consisting of spindle- and polygonal-shaped myoepithelial cells arranged in a myxoid background; the third mainly consists of myoepithelial cells with increased nuclear atypia arranged in a marked myxoid background [11, 17]. The typical biphasic growth structure consists of an inner layer of cuboidal, columnar epithelial cells with eosinophilic cytoplasm, and an outer layer from myoepithelial lineage composed of polygonal cells with abundant clear cytoplasm. Some literature reported occasional infiltrate of inflammatory cells in the tumor, such as macrophages, plasma cells, lymphocytes, eosinophils, and neutrophils [5, 12]. Squamous metaplasia was also described in a case [23]. Taken together, P-EMC mainly exists as endobronchial mass which requires a pathological biopsy combined with IHC for initial diagnosis. Bronchoscopy provides a convenient means to observe pathological patterns and make diagnosis. However, the pathological diagnosis may not be definitively made by bronchoscopic biopsy alone, and the diagnosis is mainly made by postoperative pathology combined with IHC.

The IHC results of the published P-EMC cases were retrospectively analyzed and summarized in Table 1. For the inner layer epithelial cells, 62 patients are positive for cytokeratin (CK) including CK, CAM5.2, AE3, AE1, MNF-116, CD10, CK7, CK903, CK8/18, AB, and CD117; a few cases are positive for EMA, P53, Vim, SPA, TTF-1, S-100, and SMA; only 1 case is positive for S-100; and 2 cases are positive for SMA. For the outer layer myoepithelial cells, 62 cases are positive for S-100, 38 cases are positive for SMA, and the other cases are positive for HHF-53, Vim, GFAP, P53, P63, P40, P27, actin, calponin, and CK including AE3, CD10, CK, CD117, and CK5/6 [5, 6, 7, 9, 11–42]. Briefly, the luminal layer is generally positive for the epithelial markers CK and EMA, while the outer layer is generally positive for S-100 and SMA immunostaining.

Taken together, the duct-like structure of P-EMC is characterized by biphasic pattern with an inner layer of cuboidal, columnar epithelial cells and an outside layer of myoepithelial cells. With a few exceptions, the epithelial cells are usually positive for CK; almost all the myoepithelial cells are positive S-100, followed by SMA. Therefore, the results of pathology and IHC have a great contribution to the diagnosis of P-EMC.

## 4. Diagnosis and Differential Diagnosis

Given the similar clinical presentation as other lung carcinomas, a definite diagnosis of P-EMC must be determined by a combination of clinical manifestations, imaging tests, histopathology, and IHC. The diagnosis mainly depends on the characteristics of pathology and IHC, including the biphasic pattern in pathology which has an inner layer immunoreactive for cytokeratin and an outer layer immunoreactive for S-100 and SMA. P-EMC is an exceptionally rare subtype of salivary gland lung tumor, therefore, the diagnosis relies heavily on the identification of myoepithelial cell components in a myxoid or chondroid background like other types of salivary gland tumors [17].

P-EMC is often misdiagnosed with other biphasic pulmonary neoplasms. Therefore, the differential diagnosis of P-EMC is challenging. On the one hand, P-EMC needs to be differentiated from cartilaginous hamartoma, pulmonary blastoma, and carcinosarcoma. For cartilaginous hamartoma, the cartilaginous component is predominant in the lesion and is well demarcated from the epithelial component that appears as encasement or invagination. Pulmonary blastomas present obvious sarcomatous stromal component and characteristic epithelial elements, which are similar to the developing fetal lung. Of note, carcinosarcoma is difficult to differentiate because of its cytological atypia, which lacks unambiguous sarcomatous stromal component. The epithelial is composed of adenocarcinoma, squamous, or anaplastic carcinoma and has no characteristic myoepithelial differentiation [17].

On the other hand, the diagnosis of P-EMC needs to be differentiated from salivary gland-type tumors (including pulmonary pleomorphic adenoma (PPA), MEC, ACC, clear cell carcinoma, myoepithelioma, myoepithelial carcinoma, mucous gland adenoma), and other tumors (including clear cell tumor, metastatic renal clear cell carcinoma, metastatic clear cell carcinoma of the thyroid, glandular carcinoid, and pulmonary adenosquamous carcinoma). PPA also contains a myxochondroid or chondroid stromal component as opposed to the biphasic structure of P-EMC; however, PPA tends to have little duct-like structure which is common in P-EMC [20]. The most common salivary gland tumors are MEC and ACC [3]. ACC originates from the cartilage-bearing bronchi and has three main growth patterns: cylindromatous, tubular, and solid [10]. The tubular pattern of ACC needs to be differentiated from EMC, which does not exist in an extensive infiltrative growth pattern and characteristic cribriform growth pattern [20]. Clear cell (“sugar”) tumor of the lung tends to be peripheral, which has a sinusoidal vascular with large, polygonal, and glycogen-containing clear cells. Primary and metastatic clear cell carcinomas both lack the typical pathology and IHC presentation of P-EMC, and metastatic renal clear cell carcinoma contains cytoplasmic lipid with or without glycogen [20]. Most of the “sugar” tumors are positive for HMB-45 and S-100, therefore, HMB-45 staining is helpful for the differential diagnosis [20]. In addition, as metastatic clear cell carcinoma of thyroid lacks myoepithelial, positive thyroglobulin staining supports the diagnosis of the tumor of the thyroid and positive chromogranin A, and neuro-specific enolase (NSE) staining supports the diagnosis of the tumor of the glandular carcinoid, but this dual differentiation structure is absent in P-EMC [21]. Pulmonary adenosquamous carcinoma is a unique tumor type with an excessive amyloid-like eosinophilic extracellular material, which shows both glandular and squamous structures [21]. Neither myoepithelioma nor myoepithelial carcinoma include the typical biphasic pattern [43]. Mucous gland adenoma lacks the characteristics of myoepithelial hyperplasia, but has dilated glands and eosinophilic colloids [15]. In addition to these salivary gland-type tumors, intrabronchial papillary tumors can be differentiated from P-EMC because of their prominent papillary structure [37].

In summary, the differential diagnosis depends on two factors: the myoepithelial proliferation and a noninvasive growth pattern [15]. Because P-EMC is a potentially malignant tumor, the differential diagnosis of P-EMC from other tumors is crucial for the diagnosis and treatment.

### 4.1. Treatment

The optimal treatment for P-EMC has not been determined. Surgical resection remains the routine treatment for P-EMC, while radiotherapy and chemotherapy are rarely used. Up to date, little is known about the genetic alterations in P-EMC; thus, no targeted therapy for P-EMC has been proposed.

In this review, we retrospectively analyze the reported treatment methods of P-EMC, which are summarized in Table 2 [5–7, 9, 11–42]. The current standard surgical procedures include pneumonectomy, lobectomy, sleeve lobectomy, tracheal resection, carinal resection, and wedge resection. Complete resection (R0) is defined as removal of all visible and palpable tumors, with no tumor at the margin histologically, while incomplete resection (R1 or R2) is defined as the presence of a residual tumor at the margin according to pathologic examination [28]. Lobectomy was used in most of the reported cases. In addition, treatments with nonanatomical wedge resection and curative electrosurgery were also reported. Chao et al. performed curative electrosurgery because the patient refused a pneumectomy and the tumor growth was limited to the bronchial cartilage layer [26]. In another case, Hagmeyer et al. [34] described that a patient with low-grade tumor was treated with nonanatomical wedge resection and lymph node dissection. To date, the performance of systemic lymph node dissection was only used in a few reports. Although the necessity of lymph node dissection is unclear, lymph node sampling is considered a beneficial option for tumor stage classification and prognosis of potentially aggressive P-EMC [11].

In general, lobectomy is used to treat benign manners of P-EMC which are present as small and well-defined lesions, while chemoradiotherapy is used to treat larger, invasive, or ill-defined lesions that are prone to recurrence and metastasis [17]. In previous literature, most patients did not receive subsequent radiotherapy and chemotherapy after surgery. The current radiotherapy and chemotherapy are mainly used for P-EMC patients with metastatic tumors, but only few cases have been published. For example, a case of skull metastasis received a periosteum resection and whole-brain radiation [14]. Yamazaki et al. [44] have recommended that radiotherapy should be used for EMC patients with 4 cm or larger primary tumors or positive surgical margins. As P-EMC is a low-grade malignant tumor with few recurrence or metastasis patients reported, the efficacy of chemotherapy in P-EMC is unclear. However, the incidence of lung metastases from salivary gland EMC is higher than that of P-EMC and the chemotherapy regimen for EMC with lung metastases is mainly referred to SGTTs. According to the National Comprehensive Cancer Network (NCCN), chemotherapy may be used for palliation in advanced SGTTs patients, with various agents alone or in combination (such as cisplatin, cyclophosphamide, doxorubicin, mitoxantrone, carboplatin, and vinorelbine). Yamazaki et al. [44] reported that a primary parotid EMC underwent total parotid gland resection and adjuvant radiation therapy, and a follow-up CT suggested metastasis to the lungs, with complete response (CR) of lung metastasis after 5 cycles of chemotherapy with DCF (docetaxel 60 mg/m2, cisplatin 60 mg/m2, and fluorouracil 600 mg/m2). In addition, Pierard et al. [45] described a primary submandibular gland EMC patient with lung metastases who initially treated with cisplatin+fluorouracil (CF) regimen, but due to the progression of metastases in lung, a second-line paclitaxel combined with cyclophosphamide regimen was used and eventually the disease stabilised and the lung symptoms resolved. Otherwise, EMC of the lung from the base of tongue also was reported [46]. Therefore, the current treatment of choice for patients with early-staged P-EMC is surgical resection, with follow-up treatment based on the presence or absence of high-risk factors.

Targeted therapy is indispensable in the comprehensive treatment of lung cancer and has been developing rapidly in recent years. Similar as other malignancies, EMC involves various genetic alterations including overexpression of tumor suppressor genes, inactivation of tumor suppressor genes, and deletion of certain chromosomal segments. Ru et al. [24] described a case of P-EMC with an extensive expression of the P53 gene and a possible involvement of the APC gene mutations. APC gene mutations occur in familial adenomatous polyposis and are involved in many aspects of the disease progression, but the association between APC mutation status and EMC development remains unclear. In EMC of the salivary glands, HRAS mutations are most frequently detected, followed by PIK3CA and/or AKT1 mutations [47]. HRAS mutation has not been reported for P-EMC until recently [38, 47]. Chen et al. reported a P-EMC patient with a HARS Q61R mutation, a nonsense mutation in the BCOR gene, and a TET2 C137R mutation [38]. Further studies are required to determine the clinical significance of these mutations in the BCOR and TET2 genes in the diagnosis, prognosis, and treatment of P-EMC. Unfortunately, there are currently no targeted drugs for the above mutated genes, but Mäkelä et al. [48] firstly reported an application of ex vivo drug screening together with next generation sequencing found significant differences in therapeutic sensitivity between the EMC intratumor cell lineages, and that heterogeneity of differentiation states within EMC may provide an outlet for partial therapeutic responses for targeted therapies including MEK and mTOR inhibitors.

In summary, surgical resection is still the most useful and routinely used treatment for P-EMC, whereas radiotherapy and chemotherapy are mainly used in patients with advanced diseases. The use of radiotherapy and chemotherapy mainly depends on the patient's physical condition, size of the tumor, positive margin rate, and the status of lymph node metastasis. Since genetic alterations such as HRAS mutation have been described in some recent reports, targeted therapy may also have potential utility for treatment of P-EMC. Immunotherapy has been proved effective in treatment for patients with various advanced cancers; however, the application of immunotherapy in P-EMC treatment has not been reported.

### 4.2. Prognosis

In Table 2, the disease-free survival period of P-EMC after surgery is usually more than 2 years, and even more than 5 years in some cases. Therefore, the prognosis of P-EMC is better than other NSCLC. Although P-EMC is generally considered a low-grade malignant neoplasm, it has also been shown to have the capacity to relapse and metastasize in some literature. The reported metastasis sites include hilar and subcarinal lymph node, skull, bone of left lower limb, bronchus, bilateral pulmonary, and chest wall [12–16, 27]. Cha et al. [16] summarized 6 patients with relapse or metastasis and found that no patients died of the disease, which supports the low malignant potential of P-EMC and the importance of complete tumor removal. In these metastatic or recurrent cases, resurgical resection or no treatment, the tumor did not show rapid progression, and even PFS reached more than 4 years [27, 28]. What is more, studies have suggested that size, circumscription, mitotic activity, and a myoepithelial component may be reliable clinical factors in the prognosis of P-EMC [10, 11, 15].

P-EMC has varied tumor size, ranging from 0.7 to 16 cm in diameter (average 1.9 cm) [5, 6, 7, 9, 11–42]. Nakashima et al. [11] have reviewed clinical follow-up information of 50 cases of P-EMC. They found that tumors larger than the average size are prone to metastasize and recur, whereas tumors localized in pulmonary parenchyma had no evidence of recurrence and metastasis. The mitotic activity was used to discriminate the status of recurrence and metastasis. In general, the P-EMC with no more than 1/20 high power field (HPF) and an absence of necrosis rarely recur and metastasize, while those with brisk mitotic rates (up to 13/10 HPF), necrosis and cytologic atypia indicated recurrence and/or metastasis [5].

Myoepithelial cell is an important EMC component and can be used as diagnostic and prognostic indicators. Pelosi et al. [49] suggested that abnormal intracellular localization of p27/Kip-1 in the neoplastic myoepithelial cells could abolish its growth inhibitory function, thus leading to unrestricted cell proliferation which contributes to tumorigenesis. Therefore, the myoepithelial component may have an important role in the prognosis of P-EMC. Song et al. [15] analyzed 5 P-EMC cases and found that significantly decreased nuclear expression of p27 was detected in myoepithelial cells of all the cases, which was also described in the previous three cases [7, 29, 50]. In addition, one of the P-EMC patients had a poor prognosis, mainly characterized by myoepithelial differentiation, larger size, and parenchymal invasion [15]. Furthermore, studies have also shown that simple myoepithelial tumor cells without epithelial tumor cells, such as myoepithelioma and myoepithelial carcinoma, have a poor prognosis [5, 50]. Based on the above information, myoepithelial cells may be an essential prognostic factor for P-EMC.

P-EMC is similar to the salivary gland-type EMC, of which incomplete excision is associated with poor prognosis [11]. Nakashima et al. indicated that complete resection of the tumor is needed and a thorough follow-up over 3 years is necessary after surgery [11].

As shown in Table 2[5, 6, 7, 9, 11–42], in most of the P-EMC cases, the Ki-67 index is less than 10%, indicating that the proliferation activity of the tumor cells is low, in accordance with low-grade malignant neoplasm. No metastasis event was found in the cases with a higher Ki-67 index (>10%). Therefore, there is no evident correlation between the Ki-67 index and the prognosis of P-EMC.

Although several risk factors for P-EMC prognosis have been reported as being reviewed above, further validation studies are still required. Furthermore, the clinical utility of additional prognostic features, such as the impact of radiotherapy, chemotherapy, and other treatment methods, should be assessed.

## 5. Conclusion

P-EMC is a rare subtype of SGTT, and is considered as a low-grade malignant neoplasm with relatively good prognosis. P-EMC originates from submucosal tracheobronchial glands and mainly presents as intrabronchial masses. It does not have the characteristic clinical symptom, mainly by the bronchial obstruction symptom. The diagnosis is based on the biphasic structure with an inner layer of epithelial cells immunoreactive for CK and EMA and an outside layer of myoepithelial cells immunoreactive for S-100 and SMA. Treatment of P-EMC is rarely reported. There are no specific guidelines for the treatment of P-EMC. The current treatment is mainly surgical resection, and radiotherapy and chemotherapy are mainly used in cases with poor prognosis and advanced patients. Recently, research has been started on gene mutation of P-EMC, among which the HRAS gene mutation was reported recently. Therefore, the targeted therapy for HRAS gene would be a huge step forward for P-EMC treatment. However, because of the rarity of P-EMC, the gene mutation related to P-EMC is still uncertain and needs to be studied extensively. At the same time, there is no report on immunotherapy of P-EMC. In general, size, circumscription, mitotic activity, and myoepithelial component appear to be the clinical factors that most reliably correlated with prognosis for the P-EMC. In summary, the current information about P-EMC is just the tip of the iceberg, so further study is required to acquire more precise information about P-EMC.

## Figures and Tables

**Table 1:** Clinical data of P-EMC cases.

Ref.	Age (y)/Sex	Size (cm)	Location	Smoking	Obstructive symptoms	Immunohistochemical data	
						Inner cell	Outer cell
[5]	Average 55.5 (54-57)/M (1), F (3)	Median 1.5 (1.5-5.0)	E	NA	+(3) NA (1)	EMA(+); MNF116(+); and CD34(-)	S-100(+); SMA(+); and vim(+)
[6]	51/F	3.3	P	NA	-	EMA(+); TTF-1(+)	S-100(+); actin(+)
[7]	76/F	2.7	P, U	NA	-	CK(+); EMA(+); CEA(+); and TTF-1(+)	S-100(+); actin(+); P63(+); CD10(+); and P27/kip-1(+)
[9]	7/M	3.6	E	-	-	CK(+); AE1/3(+); and S-100(-)	S-100(+); SMA(+)
[11]	54/F	1.5	E	+	-	CK7(+)	S-100(+); SMA(+); P63(+); and CK5/6(+)
[12]	Average 56.6 (52-62)/M (0), F (5)	Median 1.2 (0.6-2.6)	P	NA	+(1) -(4)	EMA(+); CK7(+); CK20(-); SPA(+); CAM5.2(+); and TTF-1(+)	S-100(+); SMA(+); CK(+); P63(+); and calponin (+)
[13]	Average 49.6 (38-56)/M (3), F (2)	Median 3 (2.5-5)	E	NA	-	CK(+); S-100(-); and SMA(-)	S-100(+); SMA(+); CK(+); CD117(+); and GFAP(+)
[14]	81/M	0.4	E	NA	-	CK(+)	S-100(+); P63(+); TTF-1(-); MIB-1(10%); and p27/kip-1(+)
[15]	Average 60.4 (52-66)/M (3), F (2)	Median 1.8 (0.7-12)	E	+(3)	+(4) -(1)	CK(+); TTF-1(+)	S-100(+); S-100(-); SMA(+); and P27(+)
[16]	53/F	2.2	E	-	+	CK(+)	S-100(+); SMA(+)
[17]	Average 52.5 (35-69)/M (2), F (6)	Median 2.5 (2.0-16.0)	E (5), P, U (3)	NA	+	CAM5.2(+); S-100(+); and CK(+)	HHF35(+); S-100(+)
[18]	55/F	2.0	E	+	+	CK(+); CAM5.2(+)	S-100(+); SMA(+); vim(+); and GFAP(+)
[19]	66/M	16	E	+	NA	CEA(+); EMA(+); and AE3(+)	S-100(+); SMA(-); AE3(+/-); and GFAP(+)
[20]	55/F	3.9	E	-	+	CK(+); GEAP(-)	S-100(+); SMA(+); and GFAP(-)
[21]	47/F	NA	E	-	+	CK(+); SMA(-)	S-100(-); SMA(+); CK(+); and vim(+)
[22]	67/M	1.3	E	+	+	CAM5.2(+); AE1/3(+)	S-100(+); SMA(+)
[23]	73/M	4.5	E	+	+	EMA(+); CK(+); S-100(-); and vim(-)	S-100(+); SMA(+); and vim(+)
[24]	73/M	3.8	E	+	+	CK(+); EMA(+); and P53(+)	S-100(+); SMA(+); GFAP(+); and P53(+)
[25]	35/F	13.0	E	NA	+	SMA(+); CD10(+)	S-100(+); SMA(+); CD10(+); and GFAP(+)
[26]	43/F	NA	E	-	+	CK7(+); EMA(+); P53(+); and CD117(+)	S-100(+); SMA(+); P53(+); and CD117(+)
[27]	74/M	1.5	E	+	+	S-100(+); SMA(+); and vim(+)	S-100(+); vim(+)
[28]	Average 57/M (1), F (1)	Median 6.9	NA	NA	NA	NA	NA
[29]	57/M	2.0	E	+	-	CAM5.2(+); AE1/AE3(+); CK7(+); and CK903(+)	S-100(+); SMA(+); GFAP(+); calponin(+); CD117(+); and P27/kip1(+)
[30]	Average 63 (36-75)/M (3), F (4)	Median 2.5 (1.3-4.0)	NA	NA	+(3)	NA	NA
[31]	34/M	1.15	E	NA	+	CK7(+); CK8/18(+)	S-100(+); SMA(+); and P63(+)
[32]	72/F	3.8	E	-	-	AE1/AE3(+)	SMA(+); P63(+)
[33]	58/M	1.3	P, U	-	-	CK(+); CEA(+); and TTF-1(+)	S-100(+); CD10(+); P63(+); and P40(+)
[34]	58/F	1.5	E	NA	-	CK7(+)	S-100(+); SMA(+); and P63(+)
[35]	83/F	8.3	E	+	+	CK(+); TTF-1(-)	S-100(+); actin(+); vim(+); and TTF-1(-)
[36]	72/M	3.5	U	NA	-	NA	NA
[37]	50/F	3.5	E	-	+	CK7(+); TTF-1(-)	S-100(+); SMA(+); P40(+); and TTF-1(-)
[38]	45/F	5.4	E	-	+	CK(+); AB(+); and TTF-1(-);	S-100(+); SMA(+); and PAS(-)
[39]	55/M	1.9	E	+	+	AE1/AE3(+); CD117(+)	S-100(+); SMA(-); P40(+); and P63(+)
[40]	30/F	NA	E	NA	+	NA	NA
[41]	76/F	2.7	E	NA	+	CK(+); vim(-)	S-100(+); P63(+); HHF(+); and CK(+)
[42]	70/F	5.0	E	+	+	CK7(+); CD117(+); CK8(+); and TTF(-)	S-100(+); SMA(+); CK5/6(+); P63(+); and P40(+)

M: male (number of people), F: female (number of people), NA: not available, E: endobronchial, P: pulmonary parenchyma, U: unrelated to the bronchial, CK: cytokeratin, EMA: epithelial membrane antigen, SMA: smooth muscle actin, GFAP: glial fibrillary acidic protein, vim: vimentin, SPA: surfactant proteins A, and TTF-1: thyroid transcription factor-1.

**Table 2:** The data on treatment and prognosis of P-EMC.

Ref.	Ki-67	Site	Treatment	Metastasis	Follow-up (month/s)
[5]	2-10%	Right main bronchus	Pneumonectomy	No	FOD: 8 mo
	1-2%	Lobe bronchus side unstated	Lobectomy	No	FOD: 60 mo
	<1%	Left main bronchus	Pneumonectomy	No	FOD: 96 mo
	1-2%	Right upper lobe bronchus	Lobectomy	No	FOD: 84 mo

[6]	NA	Left upper lobe	Lobectomy	No	FOD: 16 mo

[7]	NA	Right upper lobe	Lobectomy	No	NA

[9]	NA	Right lower segment bronchus	Lobectomy	The biphasic neoplastic cells replaced part of LN	FOD: 12 mo

[11]	<5%	Right main lower bronchus	Lobectomy	No	FOD: 36 mo

[12]	<5%	Right lower lobe	Wedge resection	NA	FOD: 31 mo
	<5%	Left lower lobe	Wedge resection	NA	FOD: 14 mo
	<5%	Right main lobe	Wedge resection	NA	FOD: 13 mo
	<5%	Left upper lobe	Wedge resection	NA	FOD: 78 mo
	<5%	Right upper lobe	Wedge resection	NA	No recurrence: 5 mo

[13]	NA	Left lower lobe	Lobectomy	1 case: infiltrated peribronchial tissue and LN metastasis	FOD: 4 mo
	NA	Right upper lobe	Lobectomy		FOD: 12 mo
	NA	Left lower lobe	Lobectomy		NA
	NA	Right upper lobe	Lobectomy		FOD: 12 mo
	NA	Left main bronchus	Pneumonectomy		FOD: 4 mo

[14]	10%	Right upper lobe bronchus	NA	Skull metastasis	NA

[15]	NA	Left lower lobe	Lobectomy	No	Recurrence
	NA	Left upper lobe	Sleeve lobectomy	No	FOD: 75 mo
	NA	Left upper lobe	Lobectomy	No	FOD: 33 mo
	NA	Right upper lobe	Lobectomy	No	FOD: 1 mo
	NA	Trachea	Endobronchial excision	No	FOD: 10 mo

[16]	<1%	Intermedius bronchus	Bilobectomy	Hilar and subcarinal LN+	Adjuvant chemotherapy

[17]	NA	Left main bronchus	Pneumonectomy	No	FOD: 72 mo
	NA	Left lower lobe	Lobectomy	No	FOD: 48 mo
	NA	Right lower lobe	Lobectomy	No	NA
	NA	Right upper lobe	Lobectomy	No	Died in the immediate postoperative period
	NA	Left upper lobe	Lobectomy	No	NA
	NA	Right lower lobe	Lobectomy	No	Recurred LN mets after 2 years
	NA	Right upper lobe	Lobectomy	No	Recurred after 3 years in trachea and right mainstem bronchus, it was treated with radiation and chemotherapy but rapidly developed mets to right cervical lymph nodes, ribs, vertebra, and left kidney and died
	NA	Left lower lobe	Lobectomy	No	NA

[18]	NA	Right upper lobe bronchus	Lobectomy	No	FOD: 24 mo

[19]	NA	Right main bronchus	Pneumonectomy	No	FOD: 36 mo

[20]	NA	Left lower basal bronchus	Lobectomy	No	FOD: 7 mo

[21]	NA	Right upper lobe bronchus	Endobronchial excision	No	FOD: 13 mo

[22]	NA	Left lower lobe bronchus	Lobectomy	No	NA

[23]	8%	Left lower lobe bronchus	Pneumonectomy	No	FOD: 34 mo

[24]	<5~20%	Left upper lobe bronchus	Lobectomy	No	FOD: 8 mo

[25]	0.1~0.5%	Right upper lobe bronchus	Pneumonectomy	No	FOD: 6 mo

[26]	2.8	Left main bronchus	Endobronchial excision	No	FOD: 6 mo

[27]	NA	Left main bronchus	Endobronchial excision	No	Recurred bilateral lung lesions

[28]	NA	Left upper lobe	Sleeve lobectomy	No	1/2 cases: recurrence
	NA	Left lower lobe	Pneumonectomy	No	

[29]	2-3%	Intermedius bronchus	Bilobectomy	No	FOD: 9 mo

[30]	NA	Right main bronchus (2)	Lobectomy (5)	Bone metastases within 3 years (1)	FOD: 60 mo (6)
	NA	Right upper lobe (1)	Pneumonectomy (1)		
	NA	Right lower lobe (2)	Sleeve lobectomy (1)		
	NA	Left upper lobe (1)			
	NA	Left lower lobe (1)			

[31]	NA	Trachea	Resection of 5 tracheal rings	NA	FOD: 24 mo

[32]	1.6%	Left basal segment bronchus	Lobectomy	No	FOD: 4 mo

[33]	NA	Left lower lobe	NA	No	FOD: 8 mo

[34]	<5%	Right upper lobe	Wedge resection	No	FOD: 24 mo

[35]	NA	Right middle lobe	NA	NA	NA

[36]	NA	Left upper lobe	Lobectomy	No	No recurrence

[37]	25%	Right middle lobe	Lobectomy	No	NA

[38]	10%	Left lower lobe	Lobectomy	No	FOD: 6 mo

[39]	NA	Right main bronchus	Electrocautery snare	NA	NA

[40]	NA	Right middle lobe	Wedge resection	No	FOD: 60 mo

[41]	NA	Left lower lobe	Endobronchial excision	NA	NA

[42]	10%	Upper trachea	Electrocautery snare	NA	NA

NA: not available, mo: month/s, LN: lymph node, FOD: free of disease, and number: number of people.
